# Cellular toxicity following application of adeno-associated viral vector-mediated RNA interference in the nervous system

**DOI:** 10.1186/1471-2202-11-20

**Published:** 2010-02-18

**Authors:** Erich M Ehlert, Ruben Eggers, Simone P Niclou, Joost Verhaagen

**Affiliations:** 1 Department of Neuroregeneration, Netherlands Institute for Neuroscience, an institute of the Royal Academy of Arts and Sciences, Amsterdam, the Netherlands; 2 NorLux Neuro-Oncology Laboratory, Centre de Recherche Public Santé, Val Fleuri, Luxembourg, Luxembourg

## Abstract

**Background:**

After a spinal cord lesion, axon regeneration is inhibited by the presence of a diversity of inhibitory molecules in the lesion environment. At and around the lesion site myelin-associated inhibitors, chondroitin sulfate proteoglycans (CSPGs) and several axon guidance molecules, including all members of the secreted (class 3) Semaphorins, are expressed. Interfering with multiple inhibitory signals could potentially enhance the previously reported beneficial effects of blocking single molecules. RNA interference (RNAi) is a tool that can be used to simultaneously silence expression of multiple genes. In this study we aimed to employ adeno-associated virus (AAV) mediated expression of short hairpin RNAs (shRNAs) to target all Semaphorin class 3 signaling by knocking down its receptors, Neuropilin 1 (Npn-1) and Neuropilin 2 (Npn-2).

**Results:**

We have successfully generated shRNAs that knock down Npn-1 and Npn-2 in a neuronal cell line. We detected substantial knockdown of Npn-2 mRNA when AAV5 viral vector particles expressing Npn-2 specific shRNAs were injected in dorsal root ganglia (DRG) of the rat. Unexpectedly however, AAV1-mediated expression of Npn-2 shRNAs and a control shRNA in the red nucleus resulted in an adverse tissue response and neuronal degeneration. The observed toxicity was dose dependent and was not seen with control GFP expressing AAV vectors, implicating the shRNAs as the causative toxic agents.

**Conclusions:**

RNAi is a powerful tool to knock down Semaphorin receptor expression in neuronal cells in vitro and in vivo. However, when shRNAs are expressed at high levels in CNS neurons, they trigger an adverse tissue response leading to neuronal degradation.

## Background

The lesion environment of the injured spinal cord constitutes an impediment to regenerating axons [1-3]. A number of neurite growth inhibitors expressed in and around the lesion area have been identified, including the myelin-associated inhibitors NogoA, myelin-associated glycoprotein (MAG), oligodendrocyte myelin glycoprotein (OMgp), EphrinB3 and Semaphorin4D as well as scar-derived factors such as CSPGs, secreted Semaphorins, Ephrins, Slits and Wnts (reviewed by Bolsover et al., Harel and Strittmatter, and Giger et al [4-6]). These proteins act through multimeric receptors expressed at the surface of injured axons. Functional interference with NogoA or its receptor stimulated the recovery of function after spinal cord lesion [6]. Neutralizing inhibitory molecules in the injured cord would be an important component of a multifaceted therapeutic strategy to promote axonal regeneration. Given the diversity of repulsive proteins, targeting of multiple ligands or their receptors will be required to produce extensive repair after CNS trauma. RNAi is a relatively new tool to silence gene expression in a sequence-specific manner. shRNAs can be used to simultaneously silence the expression of multiple genes [7-10]. We investigated whether this technology could be applied in the CNS to render injured neurons insensitive to multiple repulsive signals. As a first step in this direction we explored the feasibility to apply RNAi to interfere with the signaling of secreted chemorepulsive Semaphorins in vivo.

Semaphorins are potent chemorepulsive axon guidance cues. Secreted Semaphorins are expressed by meningeal fibroblasts invading the spinal cord lesion site [11,12]. The receptor for secreted Semaphorins is composed of a Semaphorin binding subunit (Neuropilin-1 or Neuropilin-2) and a Plexin signaling subunit (reviewed by Zhou et al. [13]). These receptors persist in corticospinal tract and rubrospinal tract (RST) neurons after injury [12,14]. Rubrospinal neurons express Npn-2 but not Npn-1. The signaling component plexinA1 and the intracellular signaling molecule CRMP2 are present in rubrospinal neurons [12]. Following injury of the RST, the expression of plexin A1 and A4 persist, whereas plexin A2 is upregulated and A3 is undetectable in the red nucleus [15]. Thus, this descending motor tract in the spinal cord is potentially sensitive to Semaphorins in the lesion core. Axon outgrowth is considerably improved when neurons are cultured on Semaphorin3A (Sema3A)-deficient meningeal cells [16] and axon crossing from an astrocyte to a meningeal cell substrate is enhanced by blocking Npn-2 [17]. Recently, an inhibitor of Sema3A was successfully used to enhance regeneration and to produce a certain degree of functional recovery of the injured spinal cord [18]. Interfering with Semaphorin-Neuropilin signaling would therefore be a promising strategy to overcome inhibition of axonal regeneration.

The potential of RNAi-based therapies as well as the utility of RNAi for basic research is widely recognized. A persistent question in the field of RNAi is how the efficiency and specificity of RNAi-mediated knockdown of gene expression can be improved. The development of RNAi has been hampered by cellular toxicity, which can be the result of interference with the endogenous miRNA machinery, the induction of innate immune responses, and off-target effects [19-24]. Here we document our attempts to block Semaphorin receptor expression by expressing shRNA molecules in neuronal cells in vitro and in the red nucleus and DRG neurons in vivo. We show that shRNA-mediated knockdown of the Semaphorin receptor Npn-1 and Npn-2 can be achieved in cultured neuronal cells by lentiviral vector derived shRNAs. One of two shRNA sequences was effective in AAV-mediated knockdown of Npn-2 in DRG neurons in vivo. Unexpectedly, AAV1-mediated expression of shRNAs in the red nucleus resulted in an adverse tissue response and neuronal degeneration.

We conclude that, although this technology has great potential to interfere with multiple inhibitory signaling pathways, the present results illustrate unanticipated problems related to the in vivo delivery of shRNA. We discuss a number of solutions that have to be implemented before this technology can be routinely applied to interfere with chemorepulsive signaling following neurotrauma.

## Results

### Efficient in vitro knockdown of Npn-1 and Npn-2 by lentiviral delivery of shRNA

**Figure 1:**
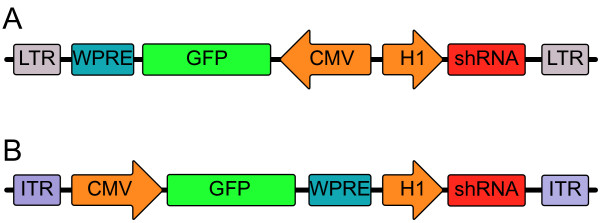
**Schematic representation of lentiviral and adeno associated constructs**. All viral particles express green fluorescent protein (GFP) under the cytomegalovirus (CMV) promoter flanked by the Woodchuck hepatitis posttranscriptional regulatory element (WPRE). In the lentiviral (LV) transfer vector (A) the shRNA expression cassette, driven by the H1 RNA promoter (H1), is placed back to back with the GFP expression cassette. In the adeno associated viral vector (AAV) the shRNA expression cassette is placed upstream of the CMV promoter (B). The packaging cassettes are flanked by inverted terminal repeats (ITR) or long terminal repeats (LTR) for the AAV and LV cassettes respectively.

**Figure 2:**
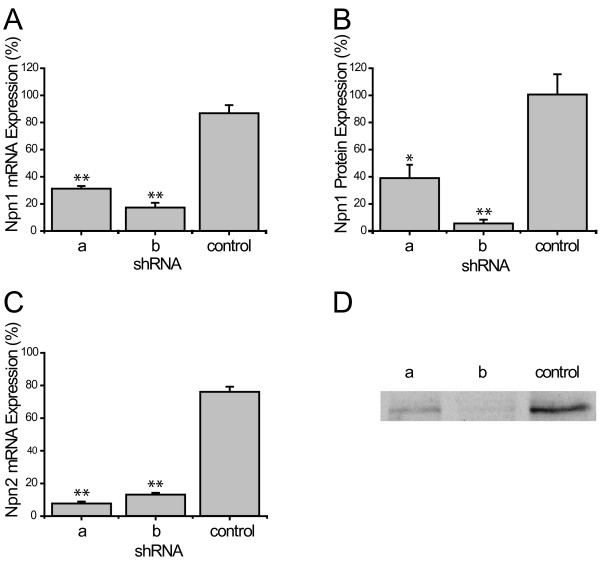
**Efficient lentivirus mediated knockdown in F11 cells**. F11 cells were infected with a lentiviral vector expressing GFP and a control shRNA and a two shRNAs directed against Npn-1 or Npn-2. Two days after infection Npn-1 mRNA expression was reduced to 31.4 ± 1.8% and 17.5 ± 3.4% (mean ± SEM). Npn-2 expression was knocked down to 7.8 ± 1.1% and 13.3 ± 1.0% (mean ± SEM) (A). Western blot (D) and quantification thereof (B) for Npn-1 showed a similar knockdown efficiency 39.2 ± 9.7% and 5.7 ± 2.6% (C) (mean ± SEM) (* <0.05, ** p < 0.005)

As a primary screening method to assess knockdown efficiency of endogenous Npn-1 and Npn-2 expression levels, F11 cells, a fusion cell line derived from of rat embryonal DRG and mouse neuroblastoma cells [25], were transduced with lentiviral vectors encoding green fluorescent protein (GFP) and an shRNAs directed against Npn-1 or Npn-2 (Figure 1a). Four days after transduction, total RNA was isolated. QPCR analysis revealed that Npn-1 expression after transduction with two shRNA sequences was significantly reduced to 31.4 ± 1.8% and 17.5 ± 3.4% respectively (Figure 2a). Western blot analysis confirmed Npn-1 knockdown at the protein level by showing that expression was reduced to 39.2 ± 9.7% and 5.7 ± 2.6% (Figure 2b,2d). Two out of seven Npn-2 shRNA sequences successfully reduced Npn-2 mRNA expression to 7.8 ± 1.1% and 13.3 ± 1.0% respectively (Figure 2c).

### AAV1-mediated overexpression of shRNA in the red nucleus results in a dose dependent adverse tissue response and neuronal degeneration

**Figure 3:**
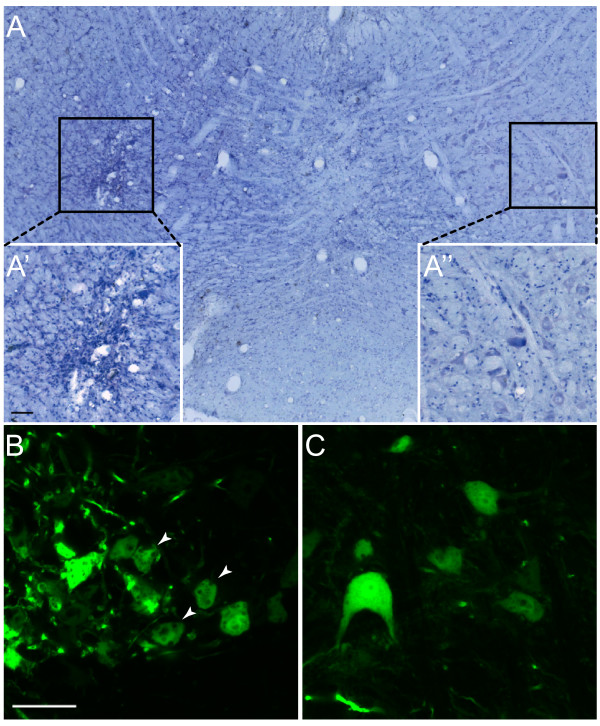
**shRNA induced toxicity in the red nucleus**. Transduction of the red nucleus with AAV1 particles expressing GFP and a control shRNA (A, B) or GFP alone (C). Injection of AAV1 particles expressing GFP and a control shRNA results in a loss of rubrospinal motor neurons and an infiltration of small diameter cells as shown in cresyl violet staining (A). Insets show enlargements of the injected (A') and contralateral side (A"). High magnification confocal microscopy images of GFP fluorescence show degenerating motor neurons (arrow heads) that were transduced with an AAV1 vector encoding shRNA (B). These degenerating neurons are not present in AAV1-GFP transduced neurons (C). Scalebar A' and B: 50 μm

**Figure 4:**
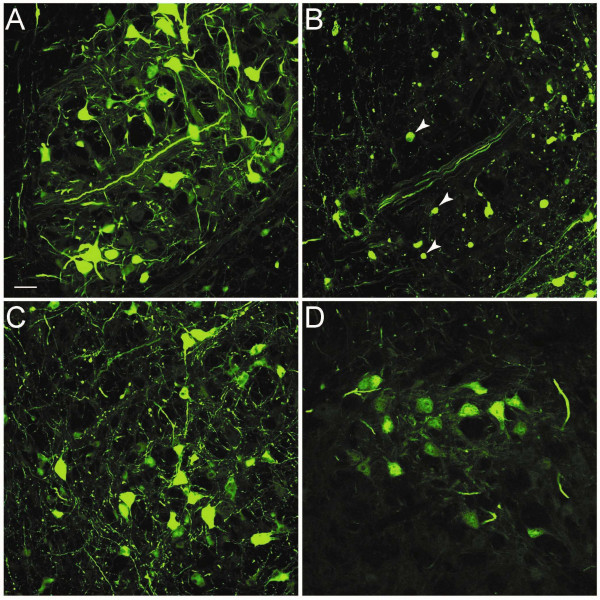
**Reduced toxicity by decreasing shRNA expression levels in the red nucleus**. GFP expressing neurons in the red nucleus shows normal morphology after injection with AAV1-GFP throughout the red nucleus (A). AAV1-shRNA injection results in profound neuronal degeneration and only occasional GFP-positive profiles of transduced neurons are observed usually at some distance from the injection site (arrow heads) (B). Lowering shRNA expression by reducing the injected AAV1 particles 10 fold (C) or using AAV2 particles (D) nearly completely alleviates toxicity. Scale bar A: 50 μm

Recent data from our lab has shown that lentiviral vectors are suboptimal transducers of rubrospinal neurons. The efficiency of transduction of red nucleus neurons is much better when using AAV1 viral particles[26]. Therefore, the control shRNA and the two shRNA cassettes that were effective in knocking down Npn-2 expression *in vitro *were cloned in the pTR-CGW AAV2 backbone (Figure 1b). After packaging, the resulting AAV1 particles mediate both GFP and shRNA expression. These vectors were stereotactically injected in the red nucleus of rats. Animals were sacrificed 3 weeks after the injection and processed for GFP-immunohistochemistry. GFP immunohistochemistry was detected in the red nucleus, demonstrating efficient transduction efficiency. However all animals injected with AAV1 shRNA vectors displayed neuronal degeneration and an adverse tissue response (Figure 3a and 3a') as compared to the uninjected contralateral nucleus (Figure 3a and 3a"). High magnification photomicrographs consistently showed atrophic morphology of neurons that were transduced with an AAV1 vector encoding shRNA (Figure 3b and 4b). This adverse tissue response and aberrant cellular morphology was not present in AAV1-GFP transduced neurons (Figure 3c and 4a), indicating that the effect was not due to GFP overexpression or AAV transduction per se.

The adverse tissue response was partially alleviated by injecting a 10 fold lower viral titer (Figure 4c). Although more neurons appear to survive under these conditions, many still have an irregular and vacuolar morphology. An alternative strategy to attenuate the level of transgene expression in rubrospinal neurons is the use of AAV2 particles. Previous experiments from our laboratory have shown that both spread and expression levels are reduced when using AAV2 as compared to AAV1 vector particles[26]. When control shRNAs were expressed by injection of AAV2 vectors in the red nucleus, GFP immunohistochemistry showed a confined population of GFP positive neurons within the red nucleus (Figure 4d). As expected, AAV2 mediated GFP expression was lower than the expression levels observed in the AAV1 injected animals and no shRNA induced adverse tissue response was present. However, after injection of AAV2 expressing Npn-2 shRNAs, no knockdown was observed under these conditions (not shown).

### AAV5 mediated knockdown of Npn-2 in rat dorsal root ganglia

**Figure 5:**
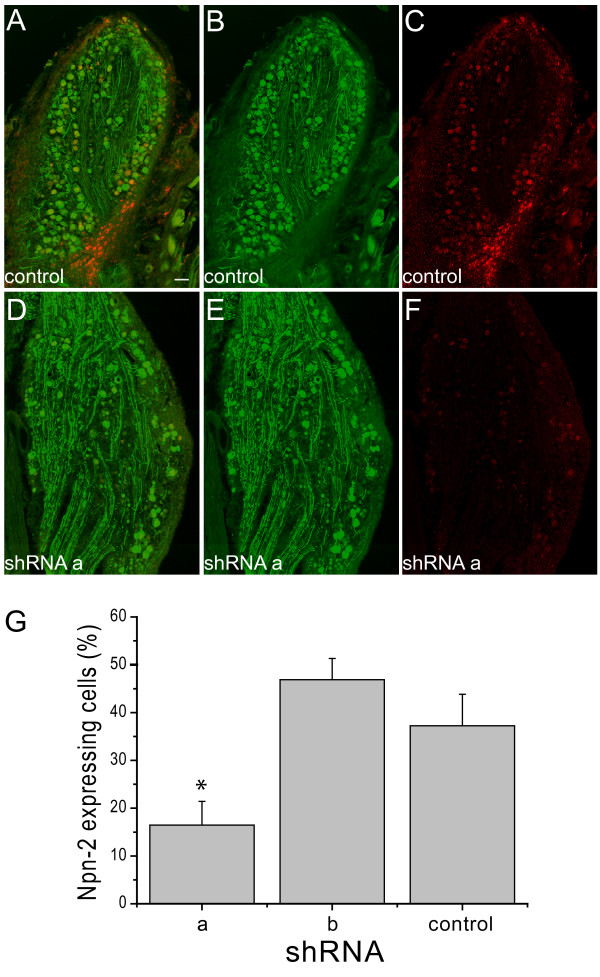
**AAV5 mediated knockdown of Npn-2 in DRG in vivo**. AAV5 particles expressing GFP and a control shRNA (A, B, C) or shRNA directed against Npn-2 (D, E, F) were injected in the L4 and L5 DRG of 3 adult female wistar rats. GFP immunoreactivity (green: A, B, D, E) and Npn-2 mRNA (red: A, C, D, F) were visualized in the same section three weeks after AAV injection. A marked reduction in the percentage of Npn-2 expressing cells among the GFP positive cells was observed after shRNA expression: 16.5 ± 5.0% versus 37.2 ± 6.6% in control animals (G) (mean ± SEM, * p < 0.05). Scalebar A: 100 μm

We also studied Npn-2 knockdown in a separate model often used for neuroregeneration studies, the rat dorsal root ganglia. Previous results showed that up to 80% of the DRG sensory neurons can be readily transduced by a single injection of AAV5 packaged viral genomes[27]. We therefore packaged our Npn-2 targeting and control shRNA vectors in AAV5 particles and injected this vector in the L4 and L5 DRG of 3 adult female Wistar rats. Three weeks after injection Npn-2 expression was analyzed by in situ hybridization. In DRGs injected with virus expressing the control shRNA 37.2 ± 6.6% of all GFP positive cells expressed Npn-2 (Figure 5a,5b,5c,5g). One of the two shRNA sequences was able to reduce the proportion of Npn-2 expressing cells to 16.5% ± 5.0 (p < 0.05) while the second shRNA was not effective (Figure 5d,5e,5f,5g). In contrast to the shRNA induced toxicity and aberrant cell morphology observed in the red nucleus, the DRG neurons appeared unaffected by the expression of shRNAs.

## Discussion

The aim of the present study was to develop an RNAi based strategy to knock down the expression of the class-3 Semaphorin receptors Npn-1 and Npn-2 in neurons of spinal nerve tracts and to employ this methodology to investigate the proposed involvement of these receptors in the failure of CNS-axons to regenerate. We have successfully developed shRNAs that knock down the expression of both Neuropilins in a neuronal cell line. In vivo, AAV5 mediated expression of the most effective Npn-2 shRNA resulted in knockdown of Npn-2 in DRG sensory neurons. Unexpectedly, AAV5-mediated expression of a second shRNA had no effect. AAV1-mediated expression of a control shRNA and a Npn-2-shRNA in the red nucleus resulted in an adverse tissue response, including neuronal cell degeneration. These observations demonstrate that, although this technology would have great potential to interfere with multiple chemorepulsive signaling pathways, unanticipated problems with cytotoxicity currently preclude the routine use of this approach in studies on neural repair in vivo.

### Selection of shRNAs for in vivo use

Despite considerable efforts to improve the selection of effective RNAi target sequences, including the development of various algorithms [28-30] and the use of favourable thermodynamic properties [31,32], several shRNA sequences against a particular target mRNA need to be screened to obtain efficient knockdown. We initially developed two shRNAs for Npn-1 and seven for Npn-2 and evaluated their capacity to silence Npn-1 or Npn-2 expression in F11 cells. Both Npn-1 and two out of seven Npn-2 shRNAs exhibit potent gene silencing following lentiviral vector-mediated delivery to the F11 cell line. When using a standard transfection for shRNA expression, most shRNA sequences are capable of reducing target expression (our own observation, not shown). The decreased effectiveness of lentiviral vector-mediated knockdown could be due to significantly lower shRNA expression levels as compared to an expression level achieved using transfection methods. Standard transfection methods deliver several hundred thousand plasmid molecules to one single cell resulting in fast-onset high level expression of shRNAs. In contrast, when shRNAs are introduced by means of lentiviral delivery, the number of genomic copies per cell is reduced by at least 3 orders of magnitude. This would result in reduced shRNA expression levels and consequent diminished knockdown efficiency of the target mRNA.

### In vivo studies

The efficacy of the selected shRNAs was tested in two neural systems that are widely used to study axonal regeneration: the sensory neurons of the DRG and the neurons of the red nucleus that form the rubrospinal tract. Work from our laboratory has shown that, as compared to AAV vectors, lentiviral vectors poorly transduce sensory neurons in vivo[27] and are suboptimal transducers of rubrospinal neurons[26]. Therefore the most effective shRNAs were expressed via AAV5 vectors in DRG and via AAV1 vectors in rubrospinal neurons. We chose these two AAV serotypes because we have shown that these are the most efficient AAV serotype vectors for these two neuronal populations [26,27].

Despite our preselection of the most effective shRNA by lentiviral vector-mediated gene silencing in F11 cells, only one of the two selected shRNAs was capable to significantly reduce the population of Npn-2 expressing DRG neurons. Although the observation that the second shRNA is somewhat less effective in vitro may already be an indication that it would be less capable to knock down Npn-2 in vivo, these observations also demonstrate that a shRNA that is effective in vitro not necessarily works in vivo.

AAV1-mediated shRNA expression in the red nucleus caused an unexpected adverse tissue reaction. Three weeks after AAV1 injection, many of the rubrospinal neurons contain vacuolar structures and have an atrophic appearance. Furthermore, there is considerable cell death as shown by the loss of neurons and the acellular granular structure of the tissue at the site of AAV injection. This phenomenon is unrelated to the shRNA sequence used and is not seen after AAV1-mediated GFP expression. It has been reported that saturating the miRNA machinery by overexpressing shRNAs, inhibits endogenous miRNA processing [22,23,33,34] with concomitant adverse effects on the transduced cells [21-24]. In the nucleus, exogenous shRNAs can saturate the function of Exportin-5, a factor required for nuclear export of pre-miRNAs and shRNAs [33-35]. This saturation can be reversed by overexpression of Exportin-5 enhancing shRNA and endogenous miRNA activity in vitro [34] and in vivo [22]. Exportin-5 expression is relatively low in brain tissue [34] as compared to other tissues, rendering the brain particularly sensitive to Exportin-5 function saturation. Similarly, in the cytoplasm, saturating the endogenous RNA induced silencing complex (RISC) may interfere with endogenous RNAi. The observation that overexpression of the catalytic RISC RNAse component Argonaut-2 enhances shRNA activity [36] demonstrates that Argonaut-2 is a rate limiting component in RNAi and is therefore prone to saturation. The cytotoxic effects observed here following AAV1-mediated delivery of shRNAs to the red nucleus appears very similar to the toxicity described before [21-23]. Saturation of the endogenous miRNA machinery may also underlie these adverse effects since lowering the viral dose, and thus the shRNA expression levels, reduced although not completely curtailed the toxic effects.

Interestingly, no toxicity was observed after AAV5-mediated delivery of shRNA to the DRG. This could be explained by our observation that, in terms of GFP expression level, AAV1-mediated expression in the red nucleus outperforms AAV5-mediated expression in the DRG. If the same holds true for shRNA expression levels, the differential expression levels could explain the difference in toxicity. Controlling the shRNA expression level by means of viral vector dose[24], regulatable promoters [37], tissue specific promoters [38] or the use of miRNAs [21,23], will be important variables for future study and in vivo application of shRNAs.

## Conclusions

Our data shows that we were able to generate shRNA sequences that efficiently knock down Npn-1 and Npn-2 expression in a neuronal cell line using a lentiviral vector delivery system. Substantial in vivo reduction of Npn-2 expression was achieved by injection of AAV5-shRNA in the DRG, without clear indication of cellular toxicity. In contrast, AAV1 mediated shRNA expression in the red nucleus triggered an adverse tissue response leading to neuronal degeneration. This cellular toxicity is likely due to high levels of shRNA expression resulting in saturation of the endogenous miRNA machinery, and has to be resolved for this technique to be routinely used in neurobiological studies.

## Methods

### Cloning and characterization of Npn-1 and Npn-2 shRNA sequences

Oligonucleotides encoding shRNAs directed against Npn-1 and Npn-2 (table 1) were cloned in the pRRLsinPPTh lentiviral (LV) vector, expressing GFP under the cytomegalovirus (CMV) promoter 3' flanked by a Woodchuck hepatitis posttranscriptional regulatory element (WPRE) and a H1 RNA polymerase promoter expression cassette (Figure 1a). As a control a shRNA lacking homology to the rat transcriptome, targeting the Arabidopsis Thaliana FUSA 5 gene, was generated (table 1). Viral particles were packaged as described before [39] using the pMD2.G and pCMVΔR8.74 packaging plasmids. F11 cells were transduced with a multiplicity of infection (MOI) of 100. Four days after transduction, protein and total RNA was isolated and Neuropilin expression levels were determined by qPCR analysis and Western Blot. The two most effective shRNA sequences were cloned in an AAV vector plasmid containing AAV2 inverted terminal repeats, a CMV-GFP expression cassette 3' flanked by a WPRE and a H1 RNA polymerase promoter expression cassette (Figure 1b).

**Table 1:** shRNA targeting sequences

Gene	Sequence
**Npn-1^a^**	cttcaacccacatttcgat
**Npn-1^b^**	gatagtaagaggtgtcatca
Npn-2	ggagtatctccaggtggac
Npn-2	gcccagccaggtgaagaat
**Npn-2^a^**	agattgtcctcaacttcaa
Npn-2	tggccggattgctaatgaa
Npn-2	catggagttccaataccaa
Npn-2	caaggagtatctccaggtgga
**Npn-2^b^**	caagcccagccaggtgaagaat
Fusa5	agatcctctgttctctctc

Targeting sequences in rat Neuropilin 1 (Npn-1) and rat Neuropilin 2 (Npn-2) genes used to generate short interfering RNAs (shRNA). **^(a,b) ^**Sequences that displayed efficient shRNA mediated knockdown of Npn-1 and Npn-2 in F11 cells when delivered by lentiviral vectors. The Arabidopsis Thaliana FUSA 5 gene (Fusa5) targeting sequence was used to create a non functional control shRNA construct.

### qPCR analysis

cDNA was synthesized using m-MLV reverse transcriptase (Invitrogen) according to manufacturer's guidelines with 500 ng total RNA and random hexamers. Npn-1 and Npn-2 expression levels were determined by quantitative PCR on an Applied Biosystems 7300 real-time PCR system using SYBR green master mix (Applied Biosystems) and 0.3 mM oligonucleotide (Eurogentec) (table 2). Gapdh, Beta-actin and Ef1-alpha expression levels were used to normalize the data for variations in cDNA input.

**Table 2:** Primers used for QPCR analysis

Gene	AccNo	Forward primer	Reverse primer
Npn-1	NM_145098	ctgtgcaaaaccaacagacctagat	gttcttgtcgcctttcccttct
Npn-2	NM_030869	tccggagagatttccatcga	aaagccgagatgggttcca
Beta actin	NM_031144	gctcctcctgagcgcaag	catctgctggaaggtggaca
Gapdh	NM_017008	tgcaccaccaactgcttagc	ggcatggactgtggtcatga
Ef1 alpha	NM_175838	accctccacttggtcgttttg	agctcctgcagccttcttgtc
CMV promoter	n.a.	aatgggcggtaggcgtgta	aggcgatctgacggttcactaa

### Western blot analysis

Cells were lysed in RIPA buffer (25 mM Tris-HCl, 150 mM NaCl, 1% Triton ×100, 1% Sodium Deoxycholate, 0.1% SDS pH 7.5). Samples were separated on 8% SDS-PAGE and transferred to a nitrocellulose membrane (Schleicher & Schuell). To detect endogenous Npn-1 a goat anti-Npn-1 antibody (1:1000, AF566, R & D systems) was used. A mouse anti-Actin antibody (1:1000, A5316, Sigma-Aldrich) was used to correct for variation in gel loading. Bands were visualized and quantified using an Odyssey Infrared Imaging Station (LI-COR) using a donkey anti-mouse-IRDye800 antibody, donkey anti-goat IRDye800 (both 1:4000, Rockland Immunochemicals) or donkey anti-mouse-Cy5 (1:400, Jackson ImmunoReseach)

### Adeno-associated viral vector preparation

Ten 9.5 cm culture dishes (Greiner Bio-One) of HEK293T cells were transfected with 50 μg AAV vector plasmid and 150 μg packaging plasmid (pDG1, pDG2 or pDG5 for AAV serotype 1, 2 or 5 respectively) [40]. The cells were harvested in lysis buffer (50 mM Tris-HCl pH 8.5, 150 mM NaCl, 2 mM MgCl_2_,1% triton-×100) 72 hrs after transfection and incubated with 10 μg/ml DNAse I (Roche) for 1 hour. Cell lysates were cleared by centrifugation at 3.200 RCF for 15 minutes and centrifuged on a step gradient containing 60, 40, 25 and 15% iodixanol (Axis Shield) for 1 h 10 m at 69.000 RPM in a Ti70 rotor (Beckman Coulter). The virus was recovered at the 40-60% interface and concentrated in Dulbecco's phosphate buffered saline (D-PBS) with 5% sucrose using a 100 kDa MWCO Amicon Ultra-15 centrifugal device (Millipore). To determine the viral titer, viral ssDNA was isolated by digesting the protein capsid with Proteinase K (Roche) and purified using MageneSil Blue beads (Promega) in SV RNA lysis buffer (Promega). Viral titers were determined by qPCR using SYBR green master mix (Applied Biosystems) and 0.3 mM primers directed against the CMV promoter (table 2).

### Experimental animals

A total of 42 adult female Wistar rats (225-250 g, Harlan) were used in this study. Animals were housed in groups under standard conditions with food and water ad libitum and a 12 h:12 h light/dark cycle. Experimental procedures were performed in accordance with the committee for laboratory animal welfare and experimentation of the Royal Netherlands Academy of Sciences.

### AAV mediated knockdown of Npn-2 in the red nucleus

The experimental groups were composed as follows: Six animals were injected with AAV1 expressing Npn-2 shRNA(a) (1.4 × 10^12 ^GC/ml), Npn-2 shRNA(b) (1.1 × 10^12 ^GC/ml) or control shRNA (1.7 × 10^12 ^GC/ml). To further study the dose dependent toxicity, 3 animals were injected with 1 μl AAV1 expressing control shRNA (1.7 × 10^12 ^and 1.7 × 10^11 ^GC/ml) or GFP only (1.7 × 10^12 ^GC/ml). To assess toxicity induced by AAV2-mediated expression of shRNAs, 6 animals were injected with 1 μl AAV2 expressing control shRNA (6.0 × 10^11 ^GC/ml). Stereotaxic injections were performed as described previously[41] under deep anesthesia with an intramuscular injection of Hypnorm (Fentanyl/Fluanisone, 0.08 ml/100 g, Janssen Pharmaceuticals) and Dormicum (Midazolam, 0.02 ml/100 g, Roche). The skull was exposed and a hole was drilled to aid positioning of the needle. A glass needle was lowered into the brain at A/P - 5.4; L+0.7; DV-6.6 from bregma. 1 μl viral vector was infused at a rate of 0.2 μl/min. Three weeks after vector injection animals were euthanized by injecting an overdose of Nembutal (sodium pentobarbital, Sanofi Sante) followed by transcardial perfusion of sequentially ice cold saline and 4% paraformaldehyde (PFA) in 0.1 M phosphate buffer.

### Injection of AAV5 in dorsal root ganglia

Per virus, three adult female Wistar rats were injected with virus expressing control shRNA (2.2 × 10^12 ^GC/ml), Npn-2 shRNA(a) (1.9 × 10^12 ^GC/ml) or Npn-2 shRNA(b) (3.9 × 10^12 ^GC/ml). Animals were deeply anesthetized by an intramuscular injection of Hypnorm (Fentanyl/Fluanisone, 0.08 ml/100 g, Janssen Pharmaceuticals) and Dormicum (Midazolam, 0.02 ml/100 g, Roche). The muscles overlaying the lumbar vertebral column were retracted and dorsal root ganglia at L4 and L5 were exposed by a partial laminectomy. 1 μl viral vector was unilaterally injected in the L4 and L5 DRG at a rate of 0.2 μl/min. Muscle layers were sutured and the skin was closed with Michell clips (Fine science tools). Three weeks after vector injection animals were euthanized by injecting an overdose of Nembutal (sodium pentobarbital, Sanofi Sante) followed by transcardial perfusion of ice cold saline followed by 4% PFA in 0.1 M phosphate-buffer.

### Immunohistochemistry and In situ hybridization

After perfusion and dissection, tissue was post fixated in 4% PFA in P-buffer, incubated in 0.25 M EDTA/PBS and cryoprotected in 25% sucrose/PBS. Tissue was embedded in OCT compound (Sakura) and snap frozen in 2-methylbutane. 20 μm Cryo sections were cut, mounted on Superfrost Plus slides (Fischer scientific) and stored at -80°C.

Immunohistochemistry staining for GFP was performed by blocking sections for one hour in Tris buffered saline (TBS) containing 0.4% Triton ×-100 and 5% fetal calf serum (FCS), followed by incubation with rabbit anti-GFP (1:100, AB3080, Chemicon) for 16 hours at 4°C. After 3 washes sections were incubated with donkey anti rabbit-Biotin (1:400, DAKO) for 3 hours in TBS, 5% FCS and 0.4% Triton-×100 followed by streptavidin-Alexa488 (1:400, Invitrogen). Fluorescent images were captured using a Zeiss LSM510 confocal laser scanning miscroscope. To visualise general brain histology, sections were counter stained with cresy violet after which bright field images were acquired using a Zeiss Axioplan microscope.

In situ hybridization was performed using digoxigenin (DIG) labelled RNA probes transcribed from rat Npn-2 cDNA as described before [42]. Antisense and sense probes were generated by in vitro transcription from linearized cDNA templates using T3 or T7 RNA polymerase (Roche) and alkali-hydrolyzed to an average length of 200 nucleotides. Slides were post-fixed in 4% PFA in PBS, digested for 10 minutes with 10 μg/ml Proteinase K (Boehringer Mannheim) in PBS containing 0.1% Triton-×100, post fixed for 15 minutes in 4% PFA in PBS and acetylated for 10 minutes in 1% triethanolamine containing 0.25% acetic anhydride. Hybridization was performed for 16 hours at 60°C in 5×Denharts, 250 μg/ml yeast tRNA, 5×SSC and 50% formamide. After stringency washes (all at 60°C, 5 minutes 5×SSC, 1 minute 2×SSC and 30 minutes 0.2×SSC, 50% formamide) slides were blocked in 1% blocking reagent (Roche) and incubated for 16 hours at 4°C with alkaline phosphatase conjugated anti-DIG fab fragments (1:3000, Boehringer Mannheim) and rabbit anti-GFP (1:100, AB3080, Chemicon) in 1% blocking agent. Secondary antibody incubations were preformed as described before. The anti-DIG antibody was visualized by incubating with Fast Red (Sigma) following manufacturers guidelines allowing color development for 16 hours at room temperature. Fluorescent images were acquired using a Zeiss Axioplan2 microscope. An algorithm was designed in Image-Pro plus (MediaCybernetics) to outline all GFP positive neurons. Per DRG, all Npn-2 positive cells were counted within this GFP positive cell population. For each experimental group (n = 6 DRGs), the average percentage of Npn-2 expression cells was calculated.

### Statistical analysis

All results are expressed as mean ± SEM. Statistical significance was tested with the student's *t*-test. A value of p < 0.05 was considered significant.

## Competing interests

The authors declare that they have no competing interests.

## Authors' contributions

JV, SPN and EME conceived the study. RE and EME carried out all technical procedures. EME, SPN and JV prepared the manuscript. All authors read and approved the final manuscript.
